# Supplementary material for: Mapping immunodominant sites on the MERS-CoV spike glycoprotein targeted by infection-elicited antibodies in humans
Source: bioRxiv. 2024 Apr 2:2024.03.31.586409. Preprint. [Version 1] doi: 10.1101/2024.03.31.586409 (PMC11014493; doi:10.1101/2024.03.31.586409)
Supplement: Supplement 1 [file NIHPP2024.03.31.586409v1-supplement-1.pdf]

bioRxiv preprint doi: https://doi.org/10.1101/2024.03.31.586409; this version posted April 2, 2024. The copyright holder for this preprint (which was not certified by peer review) is the author/funder, who has granted bioRxiv a license to display the preprint in perpetuity. It is made available under aCC-BY 4.0 International license.

Supplemental Figures

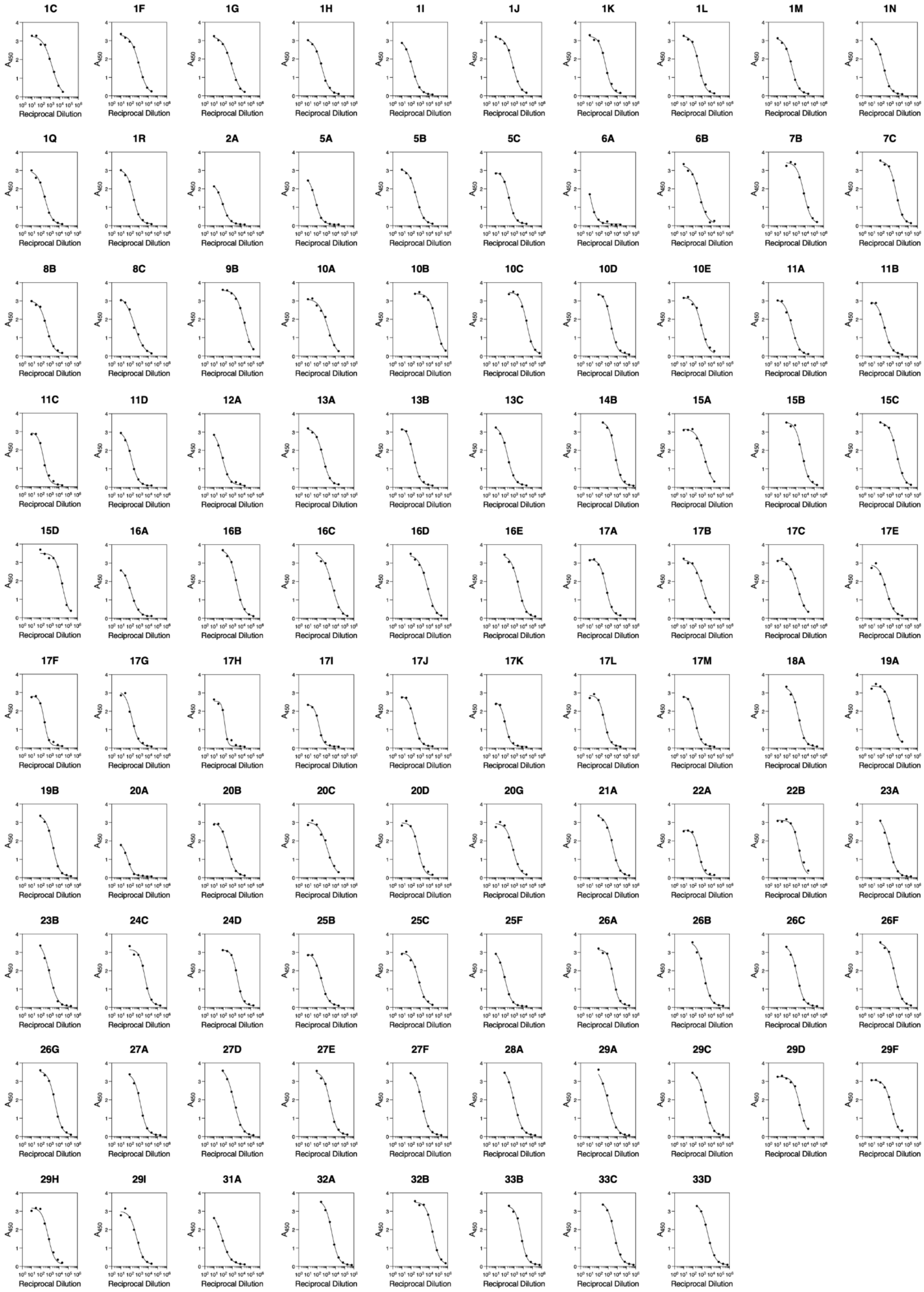

bioRxiv preprint doi: https://doi.org/10.1101/2024.03.31.586409; this version posted April 2, 2024. The copyright holder for this preprint (which was not certified by peer review) is the author/funder, who has granted bioRxiv a license to display the preprint in perpetuity. It is made available under aCC-BY 4.0 International license.

Figure S1. Evaluation of MERS-CoV S-directed plasma IgG binding titers. Dose-response curves of plasma IgG binding to prefusion-stabilized MERS-CoV EMC/2012 2P S for each of the 98 samples analyzed in this study by ELISA. Data are presented for one representative biological replicate. At least two biological replicates each using a unique batch of MERS-CoV S were conducted for each sample.

bioRxiv preprint doi: https://doi.org/10.1101/2024.03.31.586409; this version posted April 2, 2024. The copyright holder for this preprint (which was not certified by peer review) is the author/funder, who has granted bioRxiv a license to display the preprint in perpetuity. It is made available under aCC-BY 4.0 International license.

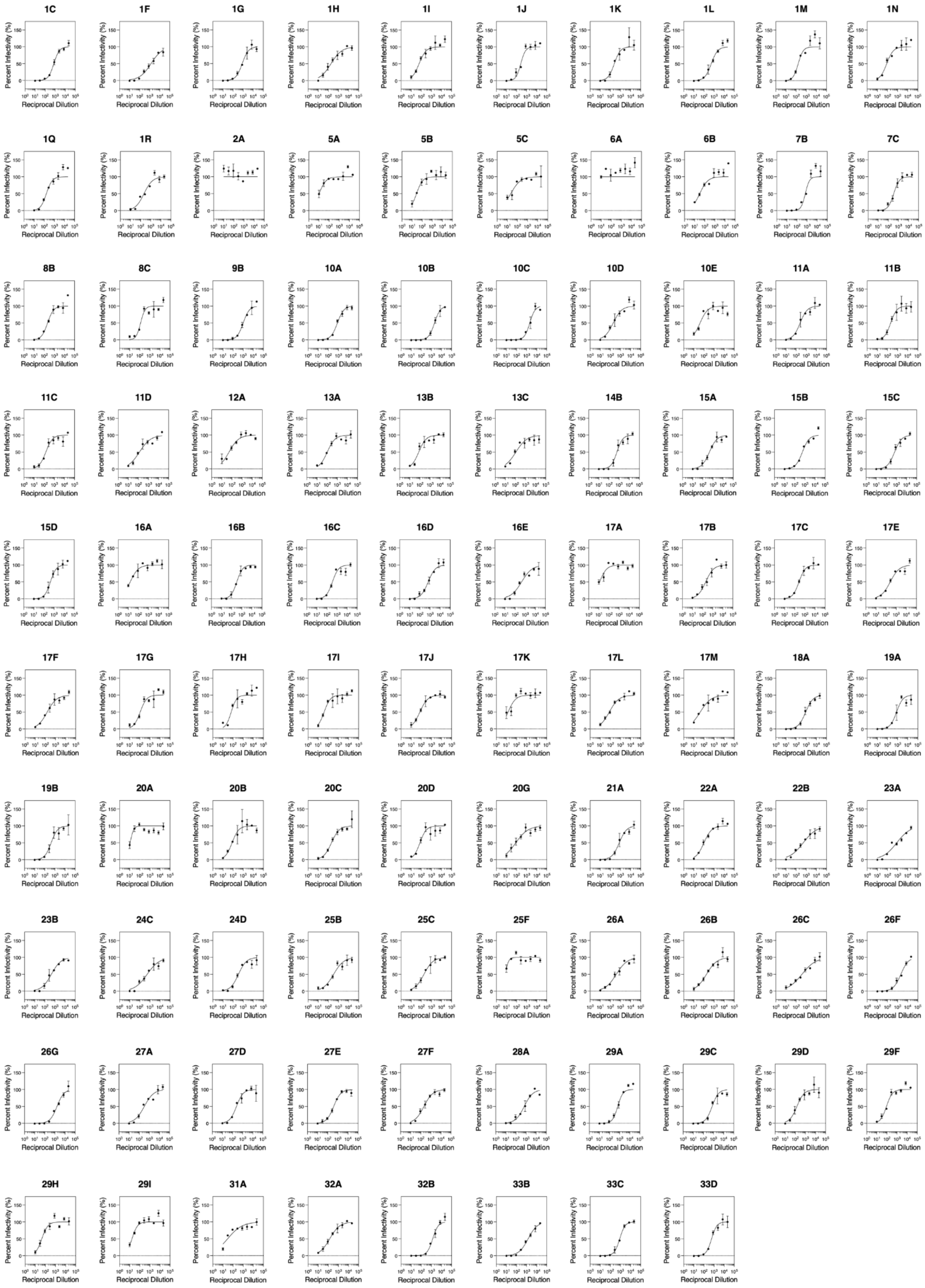

bioRxiv preprint doi: https://doi.org/10.1101/2024.03.31.586409; this version posted April 2, 2024. The copyright holder for this preprint (which was not certified by peer review) is the author/funder, who has granted bioRxiv a license to display the preprint in perpetuity. It is made available under aCC-BY 4.0 International license.

Figure S2. Evaluation of plasma neutralization potency against MERS-CoV EMC/2012. Dose-response curves of plasma neutralization of VSV pseudotyped with MERS-CoV EMC/2012 S for the 98 samples analyzed in this study. Data are presented as the mean ± standard error of two technical replicates from one representative biological replicate. At least two biological replicates with two technical replicates were completed for each sample using two distinct batches of pseudovirus.

bioRxiv preprint doi: https://doi.org/10.1101/2024.03.31.586409; this version posted April 2, 2024. The copyright holder for this preprint (which was not certified by peer review) is the author/funder, who has granted bioRxiv a license to display the preprint in perpetuity. It is made available under aCC-BY 4.0 International license.

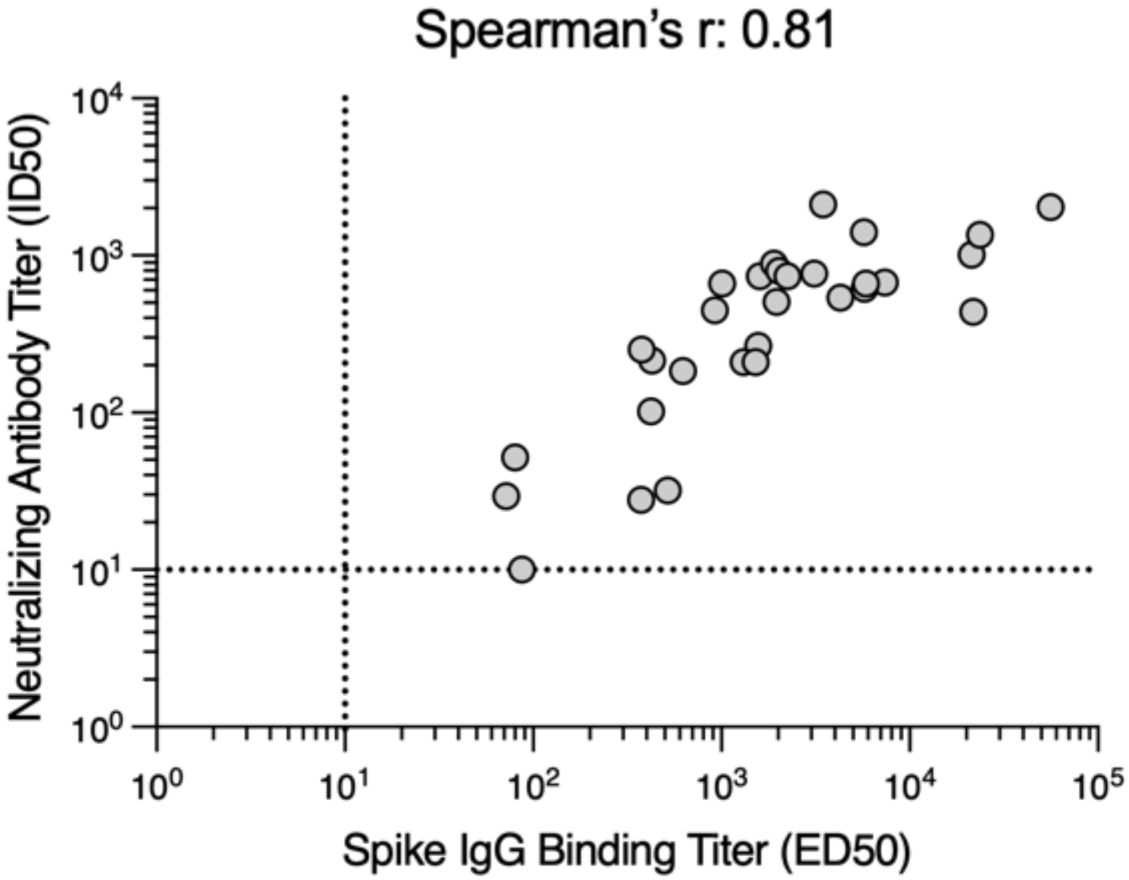

Figure S3. Correlation analysis of S-directed IgG binding titers and neutralizing antibody titers. The sample with highest S IgG binding titer per individual was included in the analysis. The limits of detection (ED_{50} or ID_{50}: ≤ 10) are indicated with dashed lines.

bioRxiv preprint doi: https://doi.org/10.1101/2024.03.31.586409; this version posted April 2, 2024. The copyright holder for this preprint (which was not certified by peer review) is the author/funder, who has granted bioRxiv a license to display the preprint in perpetuity. It is made available under aCC-BY 4.0 International license.

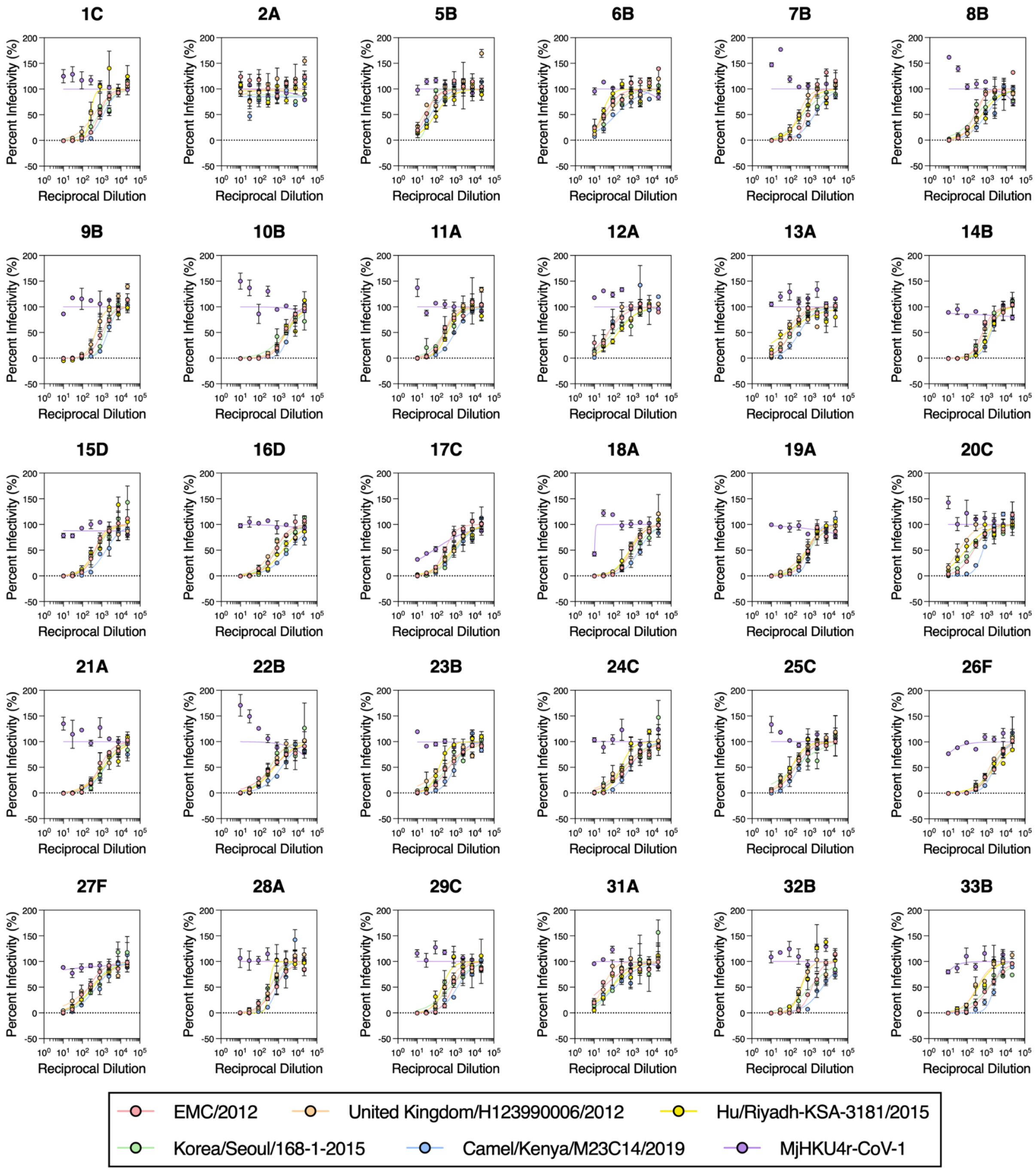

Figure S4. Evaluation of plasma neutralization potency against MERS-CoV variants and MjHKU4r-CoV-1. Dose-response curves for each of the 30 plasma samples included in the analysis using VSV pseudotyped with the indicated MERS-CoV variant or MjHKU4-CoV-1 S protein. Data are presented as mean ± standard error from one representative biological replicate. At least two biological replicates, each with two technical replicates, were conducted for each sample and each variant tested using unique batches of pseudovirus.

bioRxiv preprint doi: https://doi.org/10.1101/2024.03.31.586409; this version posted April 2, 2024. The copyright holder for this preprint (which was not certified by peer review) is the author/funder, who has granted bioRxiv a license to display the preprint in perpetuity. It is made available under aCC-BY 4.0 International license.

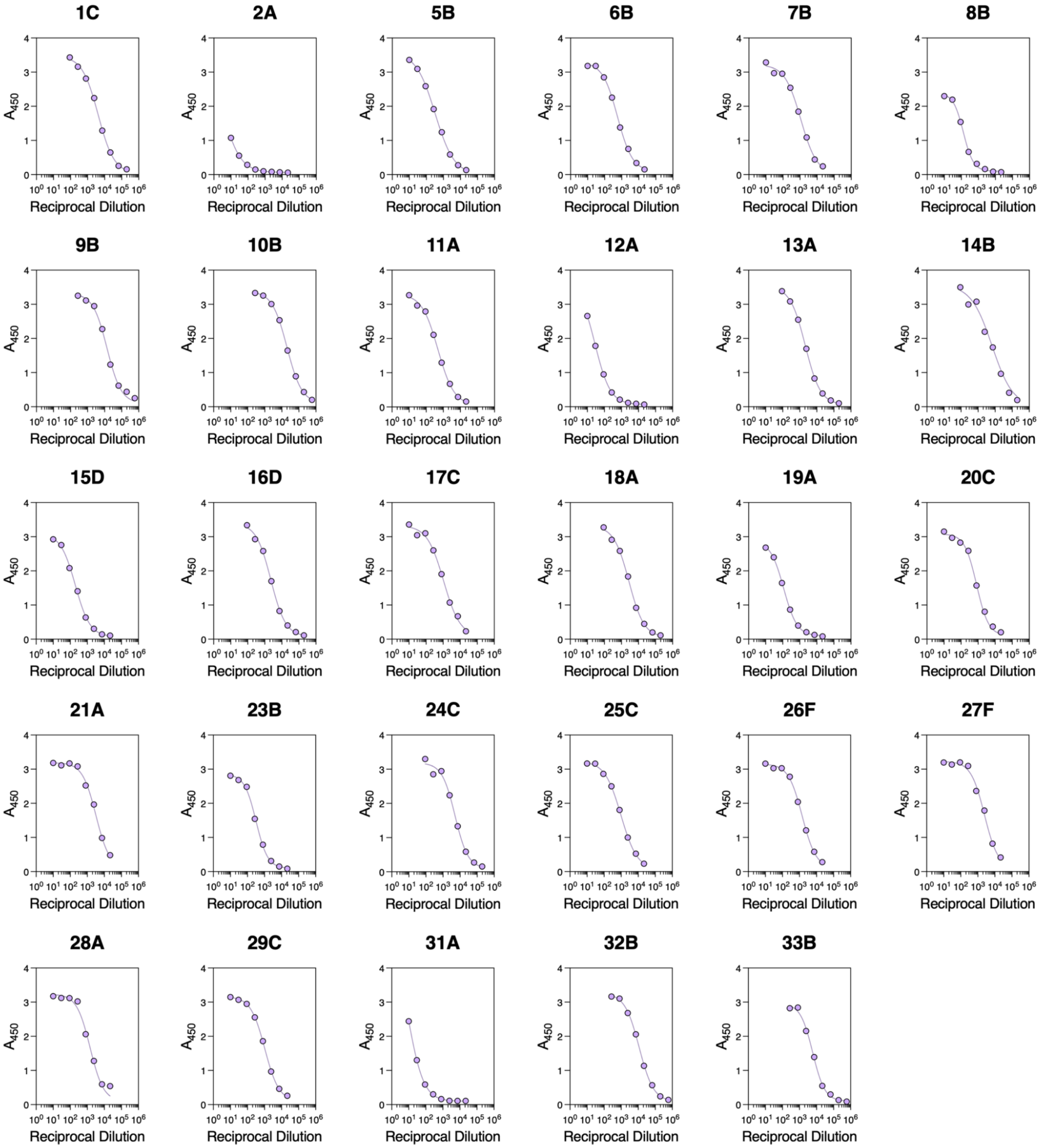

Figure S5. Evaluation of MERS-CoV S_1-directed plasma IgG binding titers. Dose-response curves of plasma IgG binding to MERS-CoV S_1 analyzed by ELISA. The sample with the highest S IgG binding titer per individual was included in the analysis. Individual 22 was excluded due to sample volume limitations. Data obtained from one representative biological replicate are presented. Two biological replicates using distinct batches of S_1 protein were completed for each sample.

bioRxiv preprint doi: https://doi.org/10.1101/2024.03.31.586409; this version posted April 2, 2024. The copyright holder for this preprint (which was not certified by peer review) is the author/funder, who has granted bioRxiv a license to display the preprint in perpetuity. It is made available under aCC-BY 4.0 International license.

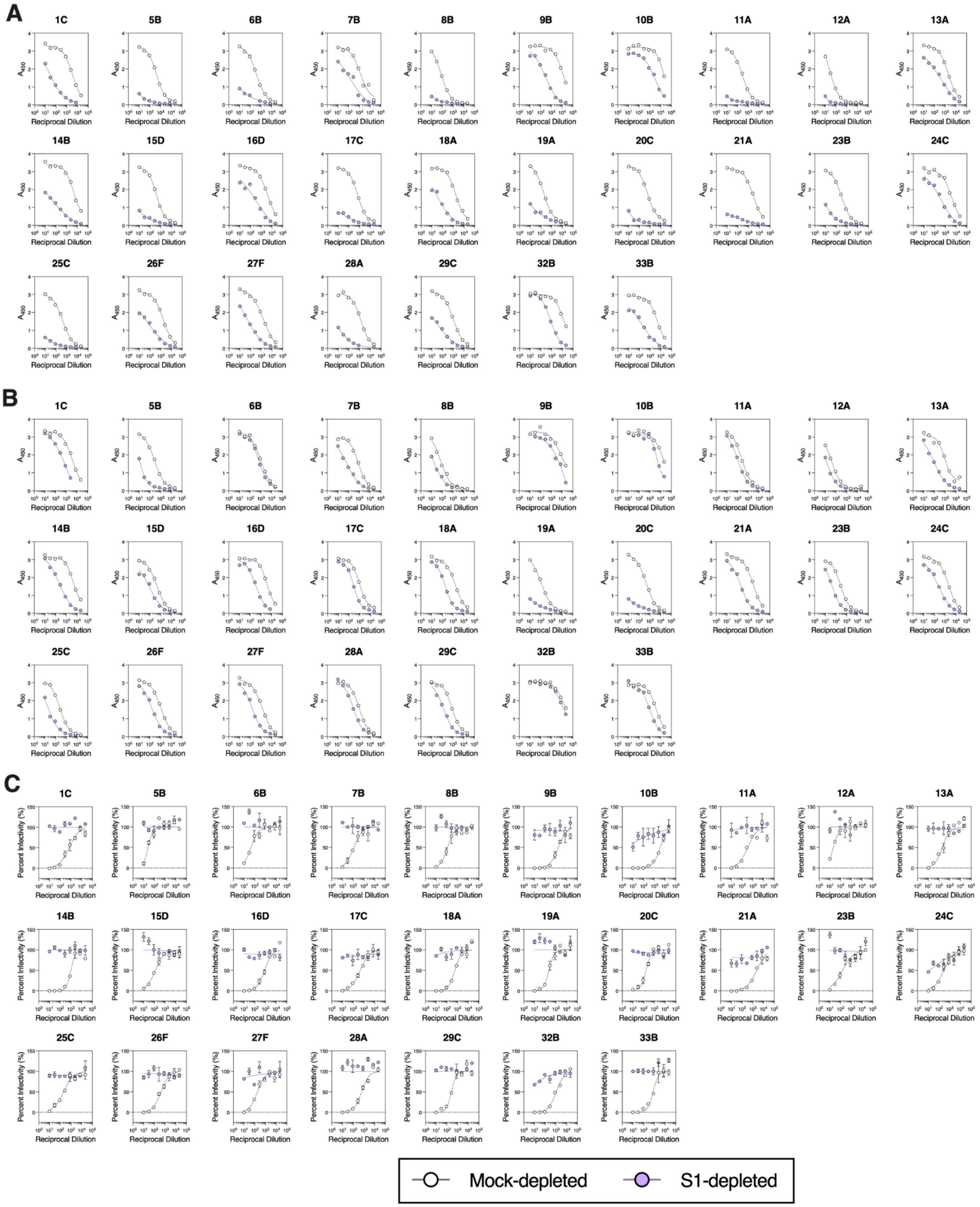

Figure S6. Evaluation of plasma IgG binding and neutralizing activity following depletion of MERS-CoV S_1-directed antibodies. A-B) Dose-response curves of S_1-(A) and S-directed (B) IgG binding for each of the 27 samples included in the analysis upon mock- or S_1-depletion analyzed by ELISA using MERS-CoV EMC/2012 S_1 or prefusion-stabilized 2P S. **C)** Dose-response curves of plasma neutralization of VSV pseudotyped

bioRxiv preprint doi: https://doi.org/10.1101/2024.03.31.586409; this version posted April 2, 2024. The copyright holder for this preprint (which was not certified by peer review) is the author/funder, who has granted bioRxiv a license to display the preprint in perpetuity. It is made available under aCC-BY 4.0 International license.

with MERS-CoV EMC/2012 S for the mock- and S_1-depleted plasma samples. Neutralization data are presented as mean ± standard error for the two technical replicates conducted. Data presented are from one representative biological replicate. Two independent biological replicates were performed using distinct batches of S_1 protein for the antibody depletions as well as unique batches of S_1 and S glycoproteins and pseudovirus for the ELISAs and neutralization assays, respectively.

bioRxiv preprint doi: https://doi.org/10.1101/2024.03.31.586409; this version posted April 2, 2024. The copyright holder for this preprint (which was not certified by peer review) is the author/funder, who has granted bioRxiv a license to display the preprint in perpetuity. It is made available under aCC-BY 4.0 International license.

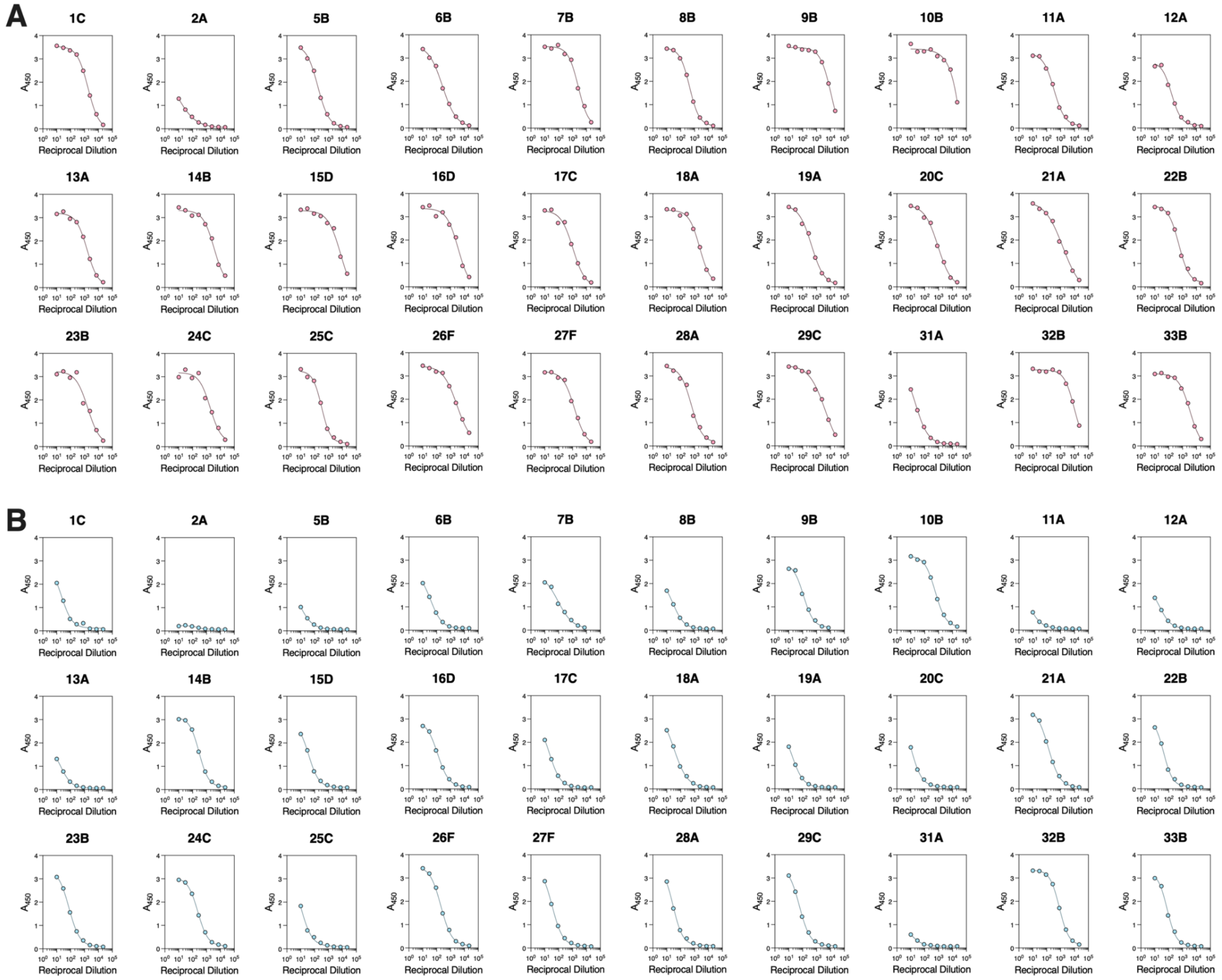

Figure S7. RBD and NTD IgG binding curves. A-B) Dose-response curves of RBD-(A) and NTD-directed (B) IgG binding for each of the 30 samples included in the analysis analyzed by ELISA using recombinantly expressed MERS-CoV EMC/2012 RBD or NTD. Data are presented from one representative biological replicate. At least two biological replicates were conducted using distinct batches of RBD or NTD.

bioRxiv preprint doi: https://doi.org/10.1101/2024.03.31.586409; this version posted April 2, 2024. The copyright holder for this preprint (which was not certified by peer review) is the author/funder, who has granted bioRxiv a license to display the preprint in perpetuity. It is made available under aCC-BY 4.0 International license.

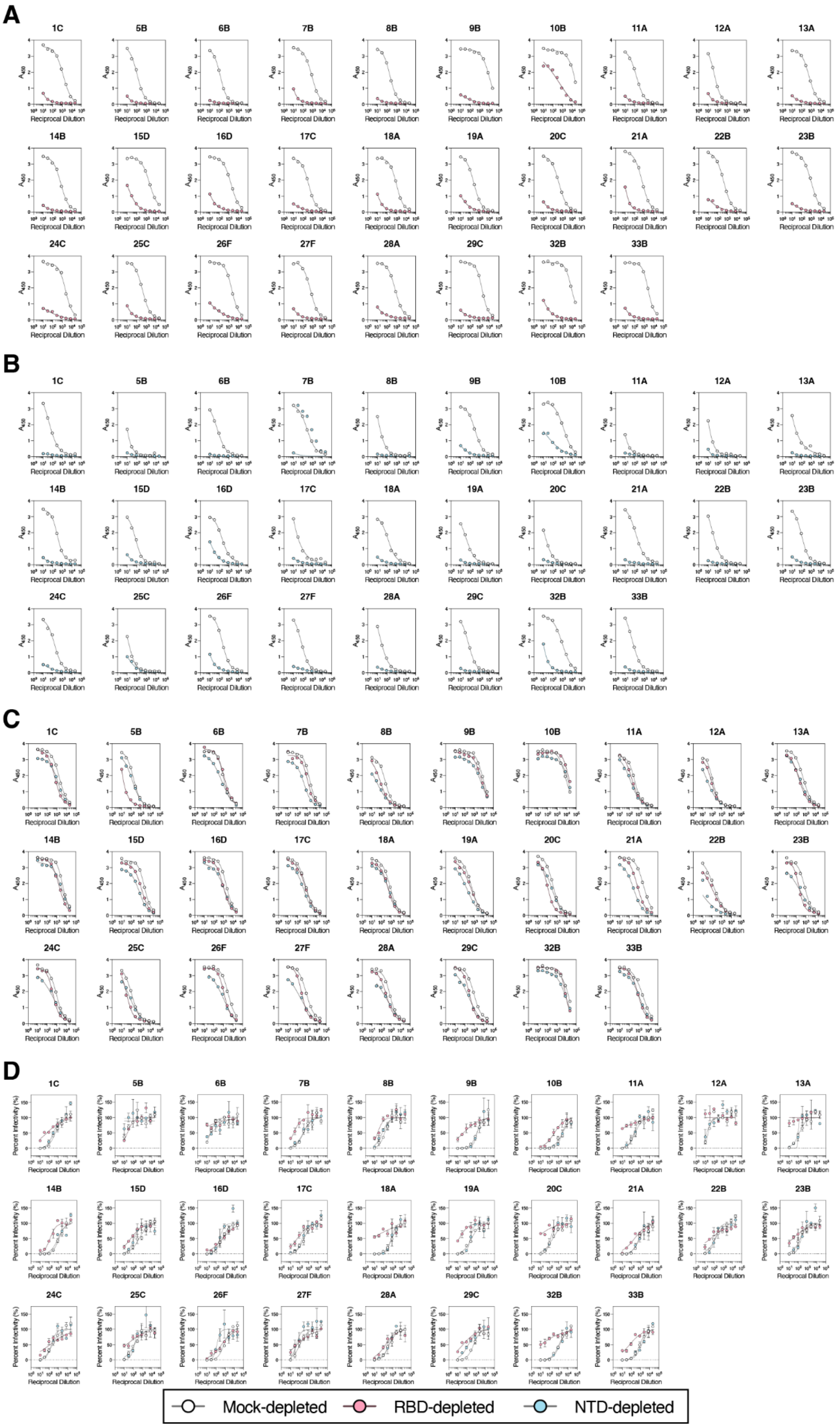

bioRxiv preprint doi: https://doi.org/10.1101/2024.03.31.586409; this version posted April 2, 2024. The copyright holder for this preprint (which was not certified by peer review) is the author/funder, who has granted bioRxiv a license to display the preprint in perpetuity. It is made available under aCC-BY 4.0 International license.

Figure S8. IgG binding and neutralization curves following depletion with MERS-CoV RBD and NTD. A) RBD IgG binding dose-response curves for each of the 28 samples included in the ELISA analysis after mock-depletion and depletion of RBD-directed antibodies. **B)** NTD IgG binding dose-response curves for each of the 28 samples included in the ELISA analysis after mock-depletion and depletion of NTD-directed antibodies. **C)** S IgG binding dose-response curves for each of the 28 samples included in the ELISA analysis after mock-depletion, depletion of RBD-directed or of NTD-directed antibodies. **D)** Dose-response curves for plasma neutralization of MERS-CoV S VSV after mock-depletion, depletion of RBD-directed or of NTD-directed antibodies. Neutralization data are presented as mean ± standard error for the two technical replicates conducted. Data presented are from one representative biological replicate. Two independent biological replicates were performed using distinct batches of RBD and NTD proteins for the antibody depletions as well as unique batches of RBD, NTD, and spike proteins and pseudovirus for the ELISAs and neutralization assays, respectively.

bioRxiv preprint doi: https://doi.org/10.1101/2024.03.31.586409; this version posted April 2, 2024. The copyright holder for this preprint (which was not certified by peer review) is the author/funder, who has granted bioRxiv a license to display the preprint in perpetuity. It is made available under aCC-BY 4.0 International license.

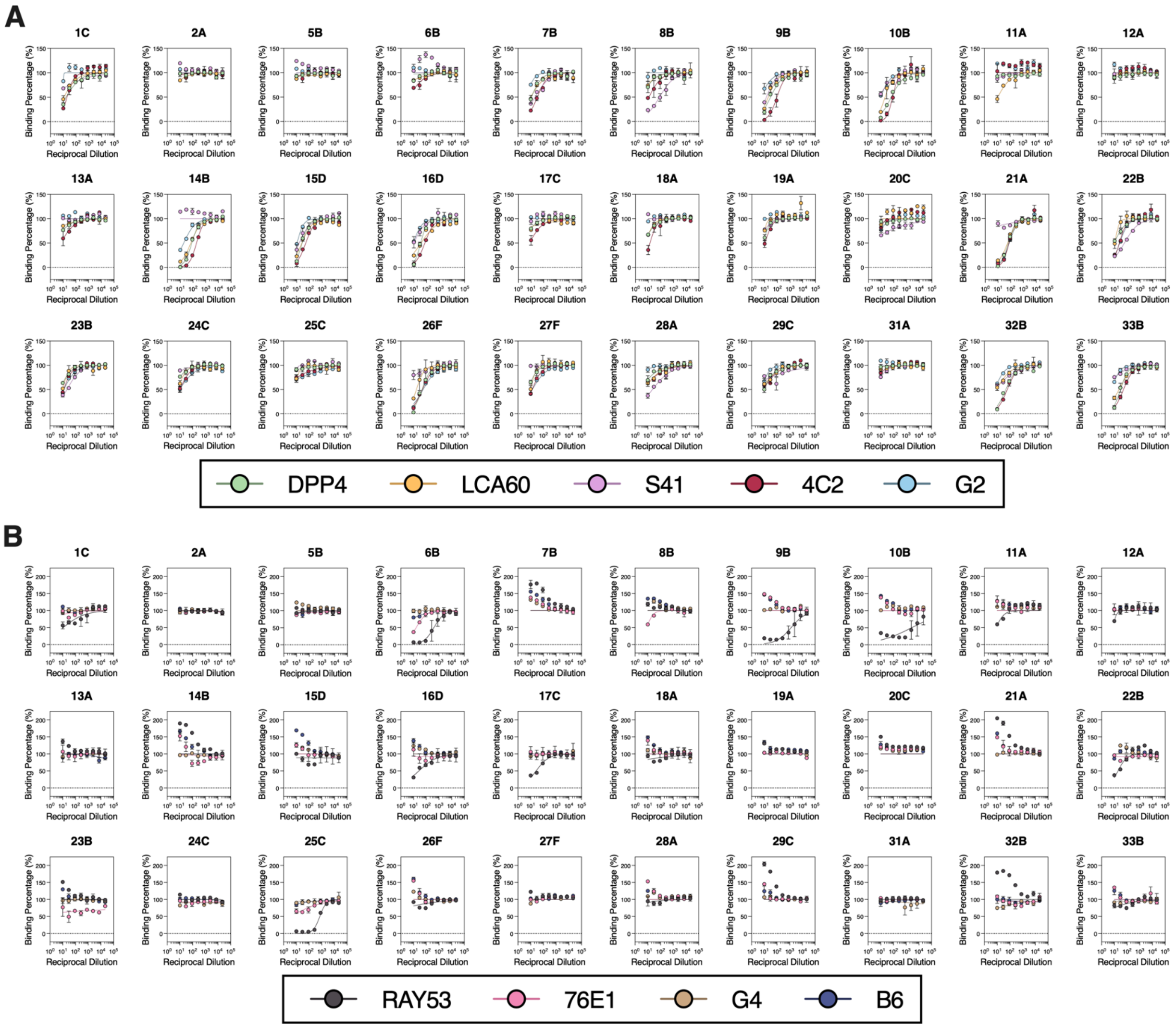

Figure S9. Competition ELISA curves. A-B) Competition ELISA curves for the 30 plasma samples analyzed using the indicated S_1-directed (A) and S_2-directed (B) monoclonal antibodies and prefusion-stabilized MERS-CoV EMC/2012 2P S . Data are presented as mean ± standard error for two technical replicates from one representative biological replicate. Two independent biological replicates using unique batches of biotinylated spike protein as well as monoclonal antibodies were performed for each sample.

bioRxiv preprint doi: https://doi.org/10.1101/2024.03.31.586409; this version posted April 2, 2024. The copyright holder for this preprint (which was not certified by peer review) is the author/funder, who has granted bioRxiv a license to display the preprint in perpetuity. It is made available under aCC-BY 4.0 International license.

Table S1. Demographic information for plasma donors.

Individual	Sex	Age	Comorbidities
1	M	42	DM, HTN
2	M	80	ESRD, DM, HTN
5	M	27	DM, HTN
6	M	64	DM, HTN
7	M	44	DM
8	M	51	DM, HTN
9	M	70	DM ,HTN, IHD
10	M	35	Psychiatric Illness
11	F	55	DM, Hypothyroidism, AKI
12	M	49	DM
13	F	41	DM
14	M	65	DM, HTN, IHD
15	M	46	DM, HTN, COPD
16	M	53	DM, HTN
17	M	49	Heart Failure
18	M	53	HTN
19	M	32	
20	M	75	DM, HTN, Epilepsy, Dyslipidemia
21	F	67	DM, HTN
22	M	39	
23	M	63	DM, BA, HTN
24	M	50	DM
25	M	59	ESRD, DM, HTN
26	M	31	ESRD, DM, HTN
27	F	70	ESRD
28	F	21	DM
29	M	69	BPH, Afib
31	F	62	ARDS, Alzheimer, DM, HTN
32	M	42	DM
33	M	58	

DM: Diabetes Mellitus; HTN: Hypertension; ARDS: Acute Respiratory Distress Syndrome; ESRD: End Stage Renal Disease; IHD: Ischemic Heart Disease; AKI: Acute Kidney Injury; COPD: Chronic Obstructive Pulmonary Disease; BA: Bronchial Asthma; BPH: Benign Prostatic Hypertrophy; A fib: Atrial Fibrillation

bioRxiv preprint doi: https://doi.org/10.1101/2024.03.31.586409; this version posted April 2, 2024. The copyright holder for this preprint (which was not certified by peer review) is the author/funder, who has granted bioRxiv a license to display the preprint in perpetuity. It is made available under aCC-BY 4.0 International license.

Table S2. Collection timepoints for the plasma samples analyzed in this study.

Individual	Sample ID	Days post symptom onset or hospitalization
1	1C	23
	1F	45
	1G	51
	1H	58
	1I	65
	1J	73
	1K	79
	1L	86
	1M	93
	1N	100
	Q	120
	1R	191
2	2A	12
5	5A	14
	5B	21
	5C	29
6	6A	6
	6B	12
7	7B	20
	7C	26
8	8B	13
	8C	23
9	9B	18
10	10A	3

bioRxiv preprint doi: https://doi.org/10.1101/2024.03.31.586409; this version posted April 2, 2024. The copyright holder for this preprint (which was not certified by peer review) is the author/funder, who has granted bioRxiv a license to display the preprint in perpetuity. It is made available under aCC-BY 4.0 International license.

	10B	9
	10C	16
	10D	23
	10E	29
11	11A	16
	11B	23
	11C	30
	11D	40
12	12A	NA
13	13A	12
	13B	19
	13C	26
14	14B	15
15	15A	12
	15B	19
	15C	26
	15D	33
16	16A	11
	16B	18
	16C	25
	16D	32
	16E	39
17	17A	5
	17B	12
	17C	19
	17E	33
	17F	40

bioRxiv preprint doi: https://doi.org/10.1101/2024.03.31.586409; this version posted April 2, 2024. The copyright holder for this preprint (which was not certified by peer review) is the author/funder, who has granted bioRxiv a license to display the preprint in perpetuity. It is made available under aCC-BY 4.0 International license.

	17G	47
	17H	54
	17I	61
	17J	68
	17K	74
	17L	82
	17M	89
18	18A	10
19	19A	6
	19B	15
20	20A	12
	20B	17
	20C	24
	20D	31
	20G	52
21	21A	7
22	22A	13
	22B	20
23	23A	7
	23B	14
24	24C	15
	24D	22
25	25B	81
	25C	88
	25F	109
26	26A	7
	26B	14
	26C	21

bioRxiv preprint doi: https://doi.org/10.1101/2024.03.31.586409; this version posted April 2, 2024. The copyright holder for this preprint (which was not certified by peer review) is the author/funder, who has granted bioRxiv a license to display the preprint in perpetuity. It is made available under aCC-BY 4.0 International license.

	26F	42
	26G	49
27	27A	10
	27D	31
	27E	38
	27F	45
28	28A	10
29	29A	10
	29C	24
	29D	31
	29F	45
	29H	59
	29I	66
31	31A	20
32	32A	5
	32B	12
33	33B	26
	33C	33
	33D	40

bioRxiv preprint doi: https://doi.org/10.1101/2024.03.31.586409; this version posted April 2, 2024. The copyright holder for this preprint (which was not certified by peer review) is the author/funder, who has granted bioRxiv a license to display the preprint in perpetuity. It is made available under aCC-BY 4.0 International license.

Table S3. Spike protein mutations in MERS-CoV variants used in this study relative to MERS-CoV EMC/2012 (NC_019843.3).

MERS-CoV Variant	Genbank Accession Number	Spike Protein Mutations
United Kingdom/H123990006/2012	NC_038294.1	L506F, Q1020H
Hu/Riyadh-KSA-3181/2015	KT806049.1	L507R, Q1020R
Korea/Seoul/168-1-2015	KT374056.1	H91Y, D510G, Q1020R
Camel/Kenya/M23C14/2019	OK094446.1	V26A, D158Y, H194Y, S390F, L450F, V597A, R626P, A1158S
